# Non-alcoholic Fatty Liver Disease in Overt Hypothyroidism: A Cross-Sectional Study in a Tertiary Care Hospital

**DOI:** 10.7759/cureus.37094

**Published:** 2023-04-04

**Authors:** Sabu Augustine, R. Harshitha, Ramesh Sangayya Hiremath, H. Anil Kumar, K. C. Prajwal

**Affiliations:** 1 Department of General Medicine, Dr. Somervell Memorial Church of South India (CSI) Medical College, Thiruvananthapuram, IND; 2 Department of General Medicine, Rajarajeswari Medical College and Hospital, Bangalore, IND; 3 Department of Medicine, Dr. Chandramma Dayananda Sagar Institute of Medical Education and Research (CDSIMER), Bangalore, IND

**Keywords:** medicine, dyslipidaemia, tsh, nafld, hypothyroidism

## Abstract

Background: The term non-alcoholic fatty liver disease (NAFLD) describes a condition in which excess fat accumulates in the liver, similar to alcohol-induced liver injury but affecting those who don't consume alcohol. Liver steatosis may vary from simple hepatic steatosis to more serious conditions, including non-alcoholic steatohepatitis and cirrhosis, and is linked to an increased risk of hepatocellular carcinoma (HCC). There is an estimated 20-30% prevalence of non-alcoholic fatty liver disease over the globe. The incidence rate among Indians is 26.9%. Metabolic diseases like insulin resistance, obesity, type-2 diabetes mellitus, and dyslipidemia are risk factors for NAFLD. A correlation between overt hypothyroidism and NAFLD has been discussed.

Objectives: To determine the magnitude of non-alcoholic fatty liver disease in overt hypothyroidism and to estimate the clinical and biochemical profile of patients with overt hypothyroidism and its relationship.

Methods: Throughout the course of a year, researchers from the medical department of a large hospital in southern India collected data in a cross-sectional observational study. Thyroid profile, fasting lipid profile, liver function tests, and ultrasound of the abdomen and pelvis were administered to a total of 100 male and female patients (18-60 years old) with newly diagnosed overt hypothyroidism who were visiting the outpatient department (OPD) and hospitalized in wards of general medicine.

Results: About 75% of subjects were females, with a mean age of 37.63±7.6 years and a mean body mass index (BMI) of 25.07±1.5 kg/m^2^. A significant correlation was found between dyslipidemia and thyroid-stimulating hormone (TSH) levels (p-value <0.001), and between dyslipidemia and ultrasonogram (USG) finding of NAFLD (p-value <0.001). A significant correlation was seen between TSH values and NAFLD findings (p-value <0.001).

Conclusion: NAFLD is a risk factor for developing hepatocellular carcinoma and is a known contributor to cryptogenic cirrhosis. Hypothyroidism is being studied as one of the causes of NAFLD. When hypothyroidism is diagnosed and treated early, it may reduce the likelihood of NAFLD and associated consequences.

## Introduction

The condition of liver lipid accumulation, resembling alcohol-induced injury but occurring in patients who do not use alcohol (or <20 g in women and <30 g in men), is called non-alcoholic fatty liver disease (NAFLD) [1]. Hepatic steatosis describes the accumulation of fat, mostly as triglycerides, cholesterol, and phospholipids, in excess of 5-10% of liver weight. Fatty liver, also known as hepatic steatosis, is one form of NAFLD, that may develop into non-alcoholic steatohepatitis (NASH) and ultimately cirrhosis of the liver [2,3]. A higher risk of developing hepatocellular carcinoma (HCC) and the subsequent need for a liver transplant are also associated with it. Twenty percent to thirty percent of the world's population may have NAFLD [4-6].

In urban parts of India, southern India has the highest prevalence (32%), followed by eastern India (24.5%), and finally western India (16.6%). Obesity, NAFLD is closely related to the development of insulin resistance, diabetes type 2, and dyslipidemia, the three hallmarks of metabolic syndrome [2,3]. Other co-morbidities include polycystic ovary syndrome, hypothyroidism, hypopituitarism, and sleep apnea. Disorders of the thyroid have also been associated with NAFLD [7]. While the incidence of hypothyroidism varies from one society to another, according to the third National Health and Nutrition Examination Survey (NHANES) survey, 4.6% of the general population has hypothyroidism [8]. Perhaps the more common endocrine disorder seen in clinical settings is hypothyroidism. It can be endemic in iodine-deficient areas but is also a common disease entity in iodine-sufficient areas. Subclinical hypothyroidism affects 4-8% of the general population, but as much as 20% of women aged 60 and more [9]. The prevalence of hypothyroidism observed in south India was 3.9%, and another 9.4% have subclinical hypothyroidism. The prevalence is higher in women, at 11.4%, in comparison with men, in whom the prevalence is 6.2% [4,10].

Thyroid hormones are crucial in the control of several metabolic pathways. In hypothyroidism, both synthesis and degradation of lipids are depressed [11]. Degradation is reduced to a greater extent than synthesis. Therefore, there is an accumulation of low-density lipoprotein (LDL) and triglycerides. A study showed a correlation between total cholesterol and serum thyroid-stimulating hormone (TSH) in hypothyroid individuals identified from among 25,862 participants, including those not aware of being hypothyroid and those on thyroid hormone replacement [11]. An increase in serum LDL has been associated with most of the studies with overt and subclinical hypothyroidism. Hypothyroidism decreases the liver's uptake of free fatty acids derived from triglycerides and is associated with a reduction in adipose tissue lipolysis [5]. NAFLD is a condition that has been linked to thyroid dysfunction, particularly overt or subclinical hypothyroidism [2]. Early detection and treatment of hypothyroidism can decrease NAFLD and its further progression [4,5].

## Materials and methods

This particular analysis was carried out at a tertiary care hospital in South India. Both male and female subjects with newly diagnosed overt hypothyroidism attending the outpatient department (OPD) and admitted to general medicine were included in the study after obtaining ethical approval from the institute with the Institutional Review Board No. RRMCH-IEC/29/2016-17. Participants ranged in age from 18 to 60, and all had clinically apparent hypothyroidism. Their whole life narrative was recorded, from their medical history and medications to their habits with tobacco and alcohol. Liver function tests, and lipid profiles are taken while they were fasting, and an ultrasound examination of the abdomen and pelvis along with other routine blood tests. There were 100 patients included in the research, and it was a cross-sectional design. Subjects included in the study were those who were newly diagnosed with overt hypothyroidism, i.e., patients with increased TSH and low T4 levels, and those with written informed permission were included.

Patients with alcohol use disorder, those who were not willing to participate in the research, subclinical hypothyroidism, diabetes mellitus, polycystic ovarian syndrome, and hepatitis B and C infections were excluded. Investigations performed included fasting lipid profile, liver function test, ultrasound of abdomen and pelvis, and thyroid profile (total T3, total T4, and TSH).

Chemiluminescent immunoassay for TSH

The quantitative analysis of human thyroid-animating chemical (hTSH) in human blood is performed by the Access hTSH assay, which is a two-site (sandwich), paramagnetic particle, chemiluminescent immunoassay. Addition of a sample to a vessel coated with goat anti-hTSH-alkaline phosphate conjugate, buffered protein solution, and a paramagnetic particle coated with immobilized mouse monoclonal anti-hTSH antibody (the mouse anti-hTSH antibody is immobilized with goat anti-mouse antibody). The serum h-TSH binds to the mouse monoclonal antibody, and the goat anti-TSH-alkaline phosphatase conjugate binds to another antigenic site of h-TSH. Separation of unbound material is done under a magnetic field and is washed off. Lumi-Phos-530, a chemiluminescent substrate, is then added to the reaction vessel, and the light generated by the reaction is measured with a luminometer. The photon production is proportional to the amount of bound enzyme conjugate, which is determined by means of a stored, multi-point calibration curve.

Cholesterol analysis

Cholesterase is an enzyme that breaks down cholesterol esters to produce cholesterol and free fatty acids. Free cholesterol, if any is present at all, is oxidized by cholesterol oxidase into cholest-4-en-3-one and hydrogen peroxide (H_2_O_2_). When H_2_O_2_ binds to 4-amino antipyrine, a chromophore (of the quinone imine kind) is formed, which is estimated at a wavelength of 505 nm.

Low-density lipoprotein (LDL) analysis

The method relies on a modified polyvinyl sulfonic acid (PVS) and polyethylene-glycol methyl ether (PEGME) coupled precipitation method, using optimized quantities of PVS/PEGME and selected detergents. LDL, very low-density lipoprotein (VLDL), and chylomicron (CM) bind to PVS and PEGME, whereas high-density lipoprotein (HDL) reacts with cholesterol oxidase (CHOD) and cholesterol esterase (CHER). The addition of detergent containing reagent (R2) releases LDL from the PVS/PEGME complex, and the released LDL reacts with enzymes to produce H_2_O_2_, which is quantified by the Trinder reaction.

High-density lipoprotein (HDL) analysis

The research relies on a modernized polyvinyl sulfonic acid (PVS) and polyethyleneglycol-methyl ether (PEGME) coupled precipitation technique with the use of optimized quantities of PVS/PEGME and chosen cleansers. LDL, VLDL, and CM bind to PVS/PEGME. CHOD and CHER selectively react with HDL to produce H_2_O_2_, which is detected through the Trinder reaction.

Triglyceride analysis

Hydrolysis of triglycerides by lipase yields free acids and glycerol. Adenosine triphosphate (ATP) is used by glycerol kinase (GK) to phosphorylate glycerol, resulting in glycerol-3-phosphate and adenosine diphosphate (ADP). Hydrogen peroxide (H_2_O_2_) produced in the Trinder reaction catalyzed by peroxidase reacts with 4-aminoantipyrine (AAP) and 4-chlorophenol (CCP) to produce a red coloration. The absorbance of this dye is proportional to the concentration of triglycerides in the sample.

Serum glutamic-oxaloacetic transaminase (SGOT) analysis

Following International Federation of Clinical Chemistry (IFCC) guidelines, the reagent is pyridoxal phosphate-free. This is a rundown of the chain of reactions that make up the assay system: L-aspartate in the sample is converted to oxaloacetate and L-glutamate by a transfer of the amino group to 2-oxoglutarate via an SGOT/aspartate amino transaminase (AST) enzyme present in the sample. In the presence of nicotinamide adenine dinucleotide dehydrogenase (NADH) and malate dehydrogenase (MDH), oxaloacetate is reduced to L-maleate, and simultaneously, NADH is oxidized to NAD. The rate of NADH oxidation to NAD is measured by tracking the reduction in absorbance at 340 nm. For the effective and quick elimination of endogenous pyruvate, which might otherwise interfere with the experiment, lactate dehydrogenase (LDH) must be added to the reagent.

Serum glutamic pyruvic transaminase (SGPT) analysis

The IFCC recommended this pyridoxal phosphate-free alanine amino transaminase/SGPT reagent. This is a rundown of the chain of reactions that make up the assay system: the amino group from alanine is transferred to the carbon atom of 2-oxoglutarate through the enzymes SGPT/alanine amino transaminase (ALAT), which are present in the sample, resulting in the formation of pyruvate and L-glutamate. With the help of the LDH in the reagent, pyruvate may be converted into lactate, while NADH is oxidized to NAD. The rate of absorbance drops at 340 nm owing to NADH oxidation is used to track the process. At the first stage of incubation, LDH quickly and fully reduces the pyruvate that naturally occurs in the sample to ensure that it does not skew the results.

For analyzing the data, the current research made use of both descriptive and inferential statistics. The results for continuous variables are shown as the mean and standard deviation (min, max), while the results for categorical variables are displayed as a number (percentage). The 5% significance threshold is used for this analysis. Data analysis programs: graphs, tables, and other visualizations were created in Microsoft Word and Excel (Microsoft Inc., USA); statistical analysis was performed in SPSS 18.0 (IBM Inc., Armonk, USA) and the R Environment, Version 3.2.2 (Hadley Wickham, USA).

## Results

The study population had a mean age of 37.63±7.66 years, with the predominant subjects being females (Table 1).

**Table 1:** Age distribution of subjects.

Age in years	No. of subjects	Percentage (%)
21-30	23	23.0
31-40	41	41.0
41-50	36	36.0
Total	100	100.0

The mean BMI of the study population was 25.07±1.5 kg/m^2^ (Table 2).

**Table 2:** BMI distribution of subjects. BMI: body mass index, kg/m^2^: kilogram per square meter.

BMI (kg/m^2^)	No. of subjects	Percentage (%)
18.5-22.9	4	4.0
23-25	47	47.0
25-30	49	49.0
Total	100	100.0

Eighty-three percent of subjects had total cholesterol >240 mg/dl. Ninety-five percent of subjects had LDL >130 mg/dl out of which 51.6% had LDL ≥190 mg/dl. Eighty-seven percent had HDL <40 mg/dl, and 99% had TG >150 mg/dl (Table 3).

**Table 3:** Lipid parameter distribution of subjects studied. mg/dl: milligrams per deciliter; LDL: low-density lipoprotein; HDL: high-density lipoprotein.

Lipid parameters	No. of subjects (n = 100)	Percentage (%)
Total cholesterol (mg/dl)		
<200	5	5
200-239	12	12
≥240	83	83
LDL (mg/dl)		
<100	2	2
100-129	3	3
130-159	21	21
160-189	25	25
≥190	49	49
HDL (mg/dl)		
<40	87	87
40-60	12	12
>60	1	1
TG (mg/dl)		
<150	1	1
150-499	99	99

Seventeen percent of subjects had SGOT >46 U/l and 25% had SGPT >49 U/l. Maximum levels of SGOT were 52 U/l and SGPT was 56 U/l. Sixty-five percent of subjects were found to have fatty liver disease on the USG of the abdomen and pelvis (Table 4).

**Table 4:** USG abdomen and pelvis findings of the subjects studied. USG: ultrasonography.

USG abdomen and pelvis	No. of subjects	Percentage (%)
Normal study	35	35.0
Fatty liver	65	65.0
Total	100	100.0

There was a significant correlation between BMI and NAFLD (p <0.001), as shown in Table 5.

**Table 5:** Comparison of BMI and NAFLD. **Highly significant. USG: ultrasonography, BMI: body mass index, kg/m^2^: kilogram per square meter, NAFLD: non-alcoholic fatty liver disease.

Variable	USG abdomen and pelvis		Total	P-value
	Normal study	Fatty liver		
BMI (kg/m^2^)	24.19±1.24	25.54±1.42	25.07±1.50	<0.001**

Patients were categorized according to WHO and Asia-Pacific guidelines of BMI as <18.5 (underweight), 18.5-22.9 (normal), 23-24.9 (overweight), and >25 (obese). There was a significant correlation between BMI and TSH levels (p-value <0.018, r-value = 0.164), as shown in Table 6.

**Table 6:** Correlation between BMI and TSH. *Significant. BMI: body mass index, kg/m^2^: kilogram per square meter, TSH: thyroid-stimulating hormone, µIU/ml: micro-international unit per milliliter.

Variable	TSH (µIU/ml)			Total	P-value
	10-50	50-100	>100		
BMI (kg/m^2^)	24.85±1.43	25.83±1.62	24.53±0.10	25.07±1.50	0.018*

The prevalence of dyslipidemia was higher in NAFLD subjects, and there was a significant correlation between TC, LDL, TG, and NAFLD (p-value <0.001), as shown in Table 7.

**Table 7:** Comparison of lipid parameters according to USG abdomen and pelvis. LDL: low-density lipoprotein; HDL: high-density lipoprotein; TG: triglycerides; **highly significant; mg/dl: milligrams per deciliter; USG: ultrasonography.

Lipid parameters	USG abdomen and pelvis		Total	P-value
	Normal study	Fatty liver		
Total cholesterol (mg/dl)	274.20±66.19	342.32±57.44	318.48±68.59	<0.001**
LDL (mg/dl)	166.83±41.80	207.26±42.79	193.11±46.47	<0.001**
HDL (mg/dl)	31.67±7.71	27.99±10.81	29.28±9.95	0.078
TG (mg/dl)	215.34±50.63	289.57±50.32	263.59±61.51	<0.001**

There was a significant correlation between TC, LDL, HDL levels, and TSH levels (p-value <0.001), as shown in Table 8.

**Table 8:** Correlation between lipid parameters and levels of TSH. LDL: low-density lipoprotein; HDL: high-density lipoprotein; TG: triglycerides; TSH: thyroid-stimulating hormone; **highly significant; mg/dl: milligrams per deciliter.

Lipid variables	TSH (µIU/ml)			Total	P-value
	10-50	50-100	>100		
Total cholesterol (mg/dl)	303.99±68.51	368.39±41.11	296.00±72.95	318.48±68.59	<0.001**
LDL (mg/dl)	177.79±40.82	242.91±24.56	186.25±45.39	193.11±46.47	<0.001**
HDL (mg/dl)	30.70±7.42	21.61±6.26	47.50±26.79	29.28±9.95	<0.001**
TG (mg/dl)	243.41±55.77	328.87±20.58	256.50±66.77	263.59±61.51	<0.001**

There was a significant correlation between TSH and NAFLD findings on USG (p<0.001). All four subjects with TSH >100 µIU/ml had USG abdomen findings of NAFLD (Tables 9, 10).

**Table 9:** Thyroid profile parameters distribution of subjects studied. Reference ranges: total T3 = 0.87-1.78 ng/ml; total T4 = 5.48-14.28 µg/dl; TSH = 0.34-5.6 µIU/ml. TFT: thyroid function test; TSH: thyroid-stimulating hormone; ng/ml: nanograms per milliliter; µg/dl: micrograms per deciliter; µIU/ml: micro-international unit per milliliter.

TFT	Number of subjects (n = 100)	Percentage (%)
T3 (ng/ml)		
<0.87	94	94.0
0.87-1.78	6	6.0
>1.78	0	0.0
T4 (µg/dl)		
<1.00	5	5.0
1.0-2.0	24	24.0
2.1-6.09	71	71.0
TSH (µIU/ml)		
10-50	73	73.0
50-100	23	23.0
>100	4	4.0

**Table 10:** Thyroid variables-distribution according to USG abdomen and pelvis. USG: ultrasonography, **highly significant, n: number, ng/ml: nanograms per milliliter, µg/dl: micrograms per deciliter, µIU/ml: micro-international unit per milliliter.

Thyroid profile	USG abdomen and pelvis		Total (n = 100)	P-value
	Normal study	Fatty liver		
T3 (ng/ml)				
<0.8	32(91.4%)	58(89.2%)	90(90%)	1.000
0.8-1.2	3(8.6%)	6(9.2%)	9(9%)	
>1.2	0(0%)	1(1.5%)	1(1%)	
T4 (µg/dl)				
<1.00	1(2.9%)	4(6.2%)	5(5%)	0.389
1.0-2.0	6(17.1%)	18(27.7%)	24(24%)	
2.1-6.09	28(80%)	43(66.2%)	71(71%)	
6.09-12.23	0(0%)	0(0%)	0(0%)	
TSH (µIU/ml)				
10-50	32(91.4%)	41(63.1%)	73(73%)	<0.001**
50-100	3(8.6%)	20(30.8%)	23(23%)	
>100	0(0%)	4(6.2%)	4(4%)	

## Discussion

In our study, out of 100 subjects with overt hypothyroidism, 75 were female. The ratio of females to males was 3:1, which was similar to the study conducted by Murgod et al. [12] about changes in electrolytes and lipid profiles in hypothyroidism, which had a female-to-male ratio of 3.1:1. The population-based study conducted in Cochin by Unnikrishnan et al. [13] showed the prevalence of hypothyroidism to be higher in women. Most of the subjects in our study were in the age group of 31 to 40 years, with the mean age being 37.63±7.66 years. It was consistent with the study conducted by Khan et al. [14] on the lipid profile in hypothyroidism, which had subjects with a mean age of 36.75±5.65 years. The mean BMI in our study was found to be 25.07±1.5 kg/m^2^. Forty-nine percent of subjects had a BMI in the range of 25-30 kg/m^2^ and 47% had a BMI in the range of 23-24 kg/m^2^. Verma et al.'s [15] study showed that thyroid dysfunction was found to be more common in obese individuals. A significant correlation was observed between an increase in levels of total cholesterol/LDL/TG and USG finding of NAFLD (Table 7; p-value <0.001), which is in accordance with a study made by Rao et al. [16]. A significant correlation was observed between an increase in SGOT/SGPT levels and NAFLD (p-value <0.001). There was a slightly higher elevation of SGPT compared to SGOT in NAFLD patients (Table 11).

**Table 11:** Comparison of liver enzymes and USG abdomen findings. USG: ultrasonography, TSH: thyroid-stimulating hormone, µIU/ml: micro-international unit per milliliter, SGOT: serum glutamic-oxaloacetic transaminase, SGPT: serum glutamate pyruvate transaminase, U/L: units per liter, **highly significant.

Variables	TSH (µIU/ml)			Total	P-value
	10-50	50-100	>100		
SGOT (U/L)	33.00±10.23	40.61±7.12	40.00±8.49	35.03±10.04	0.003**
SGPT (U/L)	34.63±12.20	44.43±8.83	45.00±7.39	37.30±12.11	0.001**

Total cholesterol, LDL, and TG were found to be significantly high, whereas HDL was found to be significantly decreased in hypothyroid individuals. There was a positive correlation between total cholesterol/LDL/TG and TSH. This was consistent with the study conducted by Khan et al. [17]. In our study, there was a significant correlation between USG abdomen findings of NAFLD and TSH levels (p-value <0.001). A significant correlation was seen between the values of liver enzymes and TSH levels. These results were consistent with the studies conducted by Ludwig et al. and Chung et al. [2,18]. According to Xu et al. [4], patients with lower free T4 (FT4) or higher TSH are more likely to develop NAFLD. Zhang et al. [19] showed that in female subjects with NAFLD, the serum TSH level was significantly higher than in controls.

In our study, obesity was seen in 49% of overtly hypothyroid individuals. The prevailing belief is that hypothyroidism causes obesity. The issue is whether or not they are causally related. Verma et al.'s [15] study showed that among obese individuals, 33% had overt hypothyroidism. It further showed that obesity was most common in overt hypothyroidism. Hypothyroidism is associated with decreased thermogenesis and decreased metabolic rate and correlates with a higher BMI and a higher prevalence of obesity [20]. Several researchers believe that shifts in TSH might be a secondary effect of obesity and that overt hypothyroidism is linked to modest weight gain. Thyrotropinemia of obesity is associated with greater vulnerability to thyroid autoimmunity and consequent hypothyroidism, which may be influenced by high leptin levels [21].

Our research has a few minor caveats. The diagnosis of fatty liver in all patients was based only on USG findings, the sensitivity and specificity of which vary from observer to observer. When the amount of fat in the liver is below 33%, it might be difficult to diagnose fatty liver disease in morbidly obese people.

TC, LDL, and TG levels in individuals with TSH between 50 and 100 µIU/ml were higher than in individuals with TSH >100 µIU/ml. This might be due to the lesser number of individuals with TSH >100 µIU/ml (four out of 100 individuals).

## Conclusions

Hypothyroidism, which is a treatable cause of NAFLD, can prevent further progression of NAFLD to cryptogenic cirrhosis and/or hepatocellular carcinoma. Dyslipidemia and NAFLD are considered to be risk factors for cardiovascular mortality and morbidity. Therefore, recognizing hypothyroidism in subjects and treating it with levothyroxine supplementation as early as possible helps in preventing the progression of NAFLD and can also control cardiovascular complications.
